# No “Self” Advantage for Audiovisual Speech Aftereffects

**DOI:** 10.3389/fpsyg.2019.00658

**Published:** 2019-03-22

**Authors:** Maria Modelska, Marie Pourquié, Martijn Baart

**Affiliations:** ^1^BCBL – Basque Center on Cognition, Brain and Language, Donostia, Spain; ^2^UPPA, IKER (UMR5478), Bayonne, France; ^3^Department of Cognitive Neuropsychology, Tilburg University, Tilburg, Netherlands

**Keywords:** speech perception, self-advantage, recalibration, adaptation, lip-reading

## Abstract

Although the default state of the world is that we see and hear other people talking, there is evidence that seeing and hearing ourselves rather than someone else may lead to visual (i.e., lip-read) or auditory “self” advantages. We assessed whether there is a “self” advantage for *phonetic recalibration* (a lip-read driven cross-modal learning effect) and *selective adaptation* (a contrastive effect in the opposite direction of recalibration). We observed both aftereffects as well as an on-line effect of lip-read information on auditory perception (i.e., *immediate capture*), but there was no evidence for a “self” advantage in any of the tasks (as additionally supported by Bayesian statistics). These findings strengthen the emerging notion that recalibration reflects a general learning mechanism, and bolster the argument that adaptation depends on rather low-level auditory/acoustic features of the speech signal.

## Introduction

Speech input is often audiovisual (AV) in nature: we hear the speaker’s voice (here referred to as auditory speech, or A) while we simultaneously see the corresponding articulatory lip-movements (here referred to as lip-read information, visual speech, or V).

Although we hardly ever see ourselves speaking, participants are better at lip-reading silent videos of themselves (i.e., “self”) than they are at lip-reading someone else (“other,” see Tye-Murray et al., 2013). Likewise, the benefit obtained from lip-read information when auditory speech is noisy is higher for “self” than for “other” (Tye-Murray et al., 2015). In both studies by Tye-Murray et al. (2013, 2015), the stimulus materials comprised previously recorded lip-read videos of “self” and “other.” Specifically, participants were recorded while producing a set of sentences. During the subsequent experiments, participants were presented with their own recordings and those obtained from other speakers either without sound (Tye-Murray et al., 2013) or with the sound masked by noise (Tye-Murray et al., 2015), and lip-read effects were quantified by asking participants which words from the sentences they recognized.

Performance after seeing “self” was compared with performance after seeing “other,” and in both studies, there was a lip-read advantage for “self.” Tye-Murray et al. (2013, 2015) framed their results within the “common coding hypothesis” that posits that “perceived events and planned actions share a common representational domain” (Prinz, 1997, page 129). Specifically, the (planned) motor actions related to producing the sentences were argued to drive the lip-read “self” advantage as “other” stimuli do not have the same representation in the motor domain as “self” sentences. However, because the stimuli comprised video recordings, the results suggest that the “self” advantage does not depend on on-line speech production, or real-time “planned actions.”

However, as mentioned earlier, the test materials in Tye-Murray et al. (2013, 2015) were sentences, which include lexical and semantic information that is generated or available during stimulus recording and experimental test. This information could be important for the lip-read “self” advantage, as such a “self” advantage was not observed in a study that used AV pseudowords (Aruffo and Shore, 2012). Aruffo and Shore (2012) administered a McGurk task in which a phonetic incongruency between the auditory and visual speech signals – such as when an /aba/ sound is combined with a lip-read /aga/ (McGurk and MacDonald, 1976) – often produces perceptual illusions (participants perceive /ada/, e.g., McGurk and MacDonald, 1976; Green et al., 1991; Sekiyama and Tohkura, 1991; Schwartz, 2010; Nath and Beauchamp, 2012; van Wassenhove, 2013; Tiippana, 2014; Basu Mallick et al., 2015; Baart et al., 2017; Alsius et al., 2018). As is typical in McGurk tasks, Aruffo and Shore (2012) asked listeners to indicate what they heard, and the critical finding was that the McGurk illusion was *weaker* for “self” than for “other.” So instead of a lip-read “self” advantage, there appeared to be a lip-read “self” *dis*advantage. The authors argued that “self” voice is presumably perceived as more reliable than “self” face, which leads to a decrease in lip-read-induced McGurk illusions for “self.” For “other,” this “self” voice advantage clearly cannot occur, so the relative influence of the lip-read signal increases, which results in more McGurk illusions for “other” (Aruffo and Shore, 2012). To sum up, in the work by Tye-Murray et al. (2013, 2015) where sentences were used, there was a *lip-read* or visual “self” advantage, whereas the McGurk task with pseudowords by Aruffo and Shore (2012) produced a voice or *auditory* “self” advantage.

Although the McGurk effect is widely used to investigate how speech perception is modulated by vision, it does not reflect natural conditions (Alsius et al., 2018). In real life, the auditory signal can be ambiguous – due to background noise or other sub-optimal listening conditions – but never fully incongruent with the lip-read information. When auditory speech is presented in noise or is phonetically ambiguous, lip-read information improves speech intelligibility (Sumby and Pollack, 1954) and “captures” perceived sound identity (Bertelson et al., 2003). Bertelson et al. (2003) presented listeners with an auditory speech pseudoword from the middle of a phonetic continuum between /aba/ and /ada/ (referred to as A?, for auditory ambiguous signal), and observed that the ambiguous sound is perceived as /aba/ when it is combined with lip-read “aba” (Vb), and is perceived as /ada/ when lip-read “ada” (Vd) is presented together with the sound. Here, we will refer to this on-line effect of lip-reading on sound perception as “immediate capture.”

Repeated exposure to such AV stimuli with ambiguous sounds can induce cross-modal learning that is observable as an auditory aftereffect. The typical procedure to assess this aftereffect, or *recalibration*, was introduced by Bertelson et al. (2003). The experimental paradigm consists of exposure – test blocks where exposure to (typically eight) repetitions of an *audiovisual* stimulus (A?Vb or A?Vd) is followed by an *auditory* test in which a small set (usually six) of ambiguous /aba/-/ada/ sounds need to be identified by the participants. The typical finding is that ambiguous sound identification after exposure to A?Vb yields more /aba/-responses than identification of the same test sound after exposure to A?Vd (e.g., Vroomen et al., 2004, 2007; van Linden and Vroomen, 2007; Vroomen and Baart, 2009, 2012; Baart and Vroomen, 2010; Baart et al., 2012).

Recalibration likely arises because the perceptual system tries to minimize the phonetic discrepancy between A and V, and not (solely) because participants are unsure about the identity of the ambiguous sound at test, and consequently base their response on the previously seen lip-read information (a visual carry-over effect). This is supported by the fact that lip-read recalibration experiments usually include exposure – test blocks where the exposure stimuli are *unambiguous* and phonetically congruent (AbVb or AdVd). Despite the fact that the lip-read information is exactly the same as in the auditory ambiguous exposure stimuli (A?Vb or A?Vd), exposure to *unambiguous* stimuli (AbVb or AdVd) produces effects in the opposite direction of recalibration: ambiguous sound identification during test now yields less /aba/-responses after exposure to AbVb than identification of the same test sound after exposure to AdVd. This contrast effect likely reflects (selective speech) adaptation (e.g., Eimas and Corbit, 1973; Diehl, 1975; Samuel, 1986), which is not a cross-modal learning effect, but an auditory-only effect that is driven by repetition of the non-ambiguous sound during exposure (Roberts and Summerfield, 1981).

In the present study, we assessed whether lip-read-induced immediate capture and recalibration and adaptation aftereffects are modulated by whether the AV stimuli contain one’s own face and voice (“self”) or someone else’s face and voice (“other”). The literature on the “self”-advantage in speech seems to be in disagreement on the issue of which modality is the source of advantage, as there is evidence for both a visual lip-read “self” advantage (Tye-Murray et al., 2013, 2015) and an auditory “self” advantage (Aruffo and Shore, 2012). As alluded to, however, these effects may (partially) be driven by the choice of stimulus materials, and using ambiguous auditory speech provides an ideal platform to assess some of the assumptions made in previous work. For example, using a McGurk paradigm, Aruffo and Shore (2012) argued that the “self” voice is more reliable than the “self” face, which leads to a reduced McGurk effect relative to “other.” If the tendency to weigh “self” voice more heavily than “self” face reflects a general mechanism, we should either not observe immediate capture or recalibration for “self,” or it should be smaller than for “other.” That is, when the percept is determined mainly by the “self” voice, participants will be unsure about identity of the ambiguous A? sound, and both immediate capture and recalibration effects will be small or non-existent. However, in A?Vb and A?Vd stimuli, the “self” voice is essentially unreliable because the speech sound is ambiguous, and it is therefore possible that participants will rely more on “self” lip-read information than in McGurk stimuli where the “self”-voice is unambiguous. If so, immediate capture and recalibration should occur for “self” when the sound is ambiguous. It is, however, not clear whether immediate capture and recalibration for “self” would then be equal to the effects for “other” (e.g., perhaps, “self” is treated as “other” because we normally do not see ourselves speaking), or would even be larger for “self” than for “other” [e.g., the lip-read “self” advantage observed for sentences – see Tye-Murray et al. (2013, 2015) – will generalize to our pseudoword stimuli because the phonetic AV conflict is smaller than in incongruent AV McGurk stimuli as used by Aruffo and Shore (2012)].

In the current study, we also included the control conditions with AV unambiguous materials that should produce adaptation effects in the opposite direction of recalibration (e.g., Samuel, 1986; Samuel and Kat, 1998; Bertelson et al., 2003; Vroomen et al., 2004; Vroomen and Baart, 2009), and again, the critical question is whether adaptation would be different for “self” and “other.” There is evidence that repeated articulation of a syllable may lead to an adaptive shift in voice onset time (Shimizu, 1977), and similar shifts for self-produced speech are observed after repeated exposure to “other” speech sounds, possibly because perception and production are rooted in a common mechanism that is fatigued by repeated auditory exposure (Cooper, 1974; Cooper and Lauritsen, 1974; Cooper and Nager, 1975). If this common mechanism is similar to the one proposed in the “common-coding hypothesis” (Prinz, 1997), adaptation to “self” voice might be stronger than adaptation to “other,” as hearing the “self” voice, but not the “other” voice, engages articulatory motor plans that strengthen the representation of the unambiguous adaptation sound. Critically, it is clear that adaptation effects for “other” that are obtained with AV stimuli are mostly – if not entirely – driven by the auditory signal, and not by the visual lip-read input (Roberts and Summerfield, 1981). In fact, adaptation is even argued to reflect a rather low-level acoustic contrast effect (Diehl et al., 1978, 1980), and it is thus possible that there is no “self” advantage in adaptation at all. More precisely, a contrast effect essentially implies that the test sound is simply perceived as being acoustically different from the exposure sound, which drives responses away from the exposure category (e.g., participants notice the acoustic difference between a clear /aba/ exposure sound and an ambiguous test sound, which results in less “b”-responses at test).

## Materials and Methods

### Participants

Sixteen native speakers of Spanish (8 males, mean age = 21.00 years, *SD* = 1.90) participated in return for a 10aaa/h payment. All participants reported to have normal hearing, had (corrected to) normal vision, and had no known neurological or language related disorders. The study was conducted in accordance with the declaration of Helsinki, and written informed consent was obtained prior to testing. The study was approved by the institute’s (i.e., the BCBL) internal Ethical committee. To facilitate the “self” vs. “other” comparison, all participants were paired with a participant with the same age and gender, resulting in a total of eight participant pairs. Both participants within a pair received the same experimental stimuli: the “self” stimuli for participant A in any given pair were the “other” stimuli for participant B in the same pair, and vice versa (i.e., “self” versus “other” was manipulated within subjects).

### Stimuli

**Figure 1:**
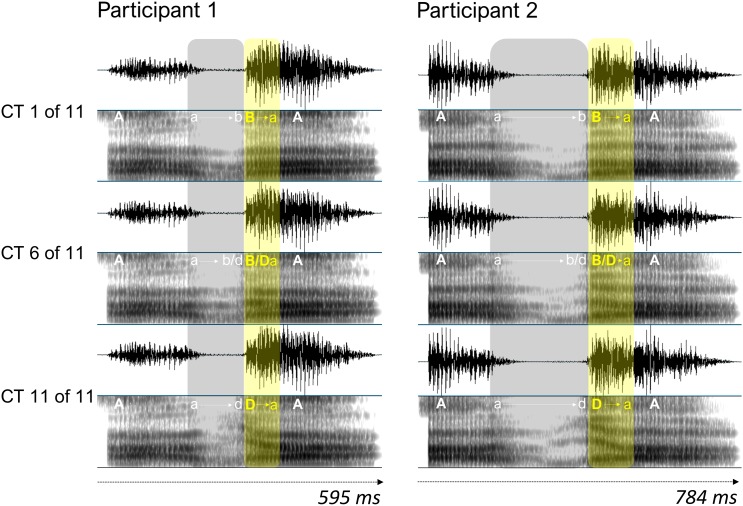
Oscillograms and spectrograms (ranging from 0 to 5000 Hz) of a male participant pair for three auditory continuum tokens (CT). Tokens 1 and 11 represent /aba/ and /ada/, respectively, and token 6 is ambiguous with respect to consonant identity. Vowels and consonants are represented in capitals, and lowercase letters and arrows represent transitions from vowel to consonant and consonant to vowel. Continuum tokens for each participant were all equal in duration, and the mean duration of the tokens across participants was 1.165 s (*SD* = 0.089). The average difference in duration between the speech sounds for participants within a pair was 96.5 ms (*SD* = 23.7).

The stimuli were created from audiovisual (AV) recordings of each participant pronouncing the pseudowords /aba/ and /ada/. Participants were instructed to pronounce multiple repetitions of the pseudowords at a natural speed, while refraining from blinking while speaking. The AV recordings (25 frames/s) were made with a digital video camera (Canon Legria HF G10) that was placed on a tripod at ∼70 cm from the participant. The video showed the participant’s full face in the middle of the screen. Stimulus preparation started with extracting the audio and video files from each recording using the FFmpeg software. Background noise in the audio files was reduced with the GoldWave and Audacity software packages, and for each participant, one /aba/ and one /ada/ stimulus – that were similar in terms of duration, intonation, loudness and pitch – were selected. In the Praat software (Boersma and Weenink, 2016), the /aba/ and /ada/ speech sounds were synthesized into an eleven-token /aba/-/ada/ auditory continuum by using a script from Matthew Winn, freely available for download^1^. The automated script allows for manual adjustments to the speech formants, and we adjusted the (variation between the) frequencies in the second (F2) and third (F3) formant tracks (where the difference between /b/ and /d/ phonemes is mainly defined) if needed (see Figure 1 for an example).

The videos that corresponded to the original /aba/ and /ada/ sounds were extracted from the recording, with a 520 ms visual lead relative to sound onset. All video clips were 1800 ms long, and converted into an image string that comprised 45 frames that were cropped to 12.4 (width) by 15.6 (height) cm in size (all cropped videos showed the entire face in the center of the frame). Whenever the end of the video contained an anomaly (such as a blink) or overlapped with a new articulation made by the participant, the corresponding frames were replaced by black images.

### Procedure

The total experiment required two sessions on two different days. On day 1, the AV recordings were made. Each participant was recorded individually during a ∼15 min session. Participants were instructed to produce /aba/ and /ada/ with similar pitch, duration, loudness and intonation. Before recording started, participants were provided with example recordings of one of the authors (MB) that served as targets.

After the stimuli were prepared, participants were invited back to the lab for the experimental session on day 2. The time in between the two sessions ranged from 12 to 33 days. In the experimental session, participants completed three subsequent tasks: an *auditory identification* task, a *recalibration/adaptation* task (testing for phonetic recalibration and selective speech adaptation), and a rating task to assess *immediate capture*. During the experimental tasks, participants were seated in a sound attenuated and dimly lit testing booth at ∼70 cm from a 17 in. CRT monitor (ViewSonic E90f, 100 Hz vertical refresh). The image strings were displayed in the middle of the screen and were surrounded by a black background. Sounds were presented at 65 dBA via two loudspeakers (JBL Duet) placed on the right and left side of the monitor. All tasks were run in PsychoPy 1.83 (Peirce, 2009), and lasted ∼50 min in total.

#### Auditory Identification

Participants received 220 trials during which they heard tokens from the continuum with their own voice (“self”), or from the continuum with the voice of the participant they were paired with (“other”). All 11 continuum tokens for “self” and “other” were presented ten times each, in random order. On each trial, a fixation cross in the center of the screen was delivered simultaneously with the sound. 1500 ms after sound onset, the letters “b” and “d” appeared left and right of the central fixation, and participants indicated whether they heard /aba/ or /ada/ by pressing a designated key on a regular keyboard (the “a” key was labeled as “b,” and the “l” key as “d”), respectively. The next trial started 500 ms after a response was collected. After data collection, the mean proportion of “b”-responses for each token was calculated, and the tokens from the “self” and “other” continua that were closest to a proportion of “b”-responses of 0.50 were considered as the participant’s most ambiguous tokens A? (henceforth referred to as A?_self_ and A?_other_ for “self” and “other,” respectively), and used during subsequent recalibration, adaptation and rating tasks.

#### Recalibration/Adaptation

Participants completed 40 AV exposure – auditory test blocks, presented in random order in a single run (∼30 min). Half of the exposure – test blocks were intended to induce *recalibration* (with ambiguous sounds), the other half were intended to induce *adaptation* (with unambiguous sounds). In all blocks, 8 repetitions of one AV exposure stimulus (ISI = 1000 ms) were followed by 6 auditory stimuli (the first auditory stimulus was delivered 500 ms after the last exposure stimulus ended). Participants were instructed to pay close attention to the exposure videos (which was monitored by the experimenter via a direct camera feed from the experimental booth) and were required to indicate whether they heard /aba/ or /ada/ on each of the 6 test sounds (ISI = 500 ms) by pressing the designated key on a regular keyboard. The test sounds were always the individually determined most ambiguous token A? and the neighboring tokens A?-1 (more “aba-like”) and A?+1 (more “ada-like”), all presented twice in pseudo-random order.

The AV exposure – auditory test blocks to induce *recalibration* contained the individually determined ambiguous A? sound that was delivered in combination with the /aba/ or /ada/ video (only one lip-read video was used in a single exposure – test block). There were 10 recalibration blocks for “self” (5 with A?_self_Vb_self_ exposure stimuli, and 5 with A?_self_Vd_self_ exposure stimuli), and 10 blocks for “other” (5 A?_other_Vb_other_ and 5 A?_other_Vd_other_ blocks). The AV exposure – auditory test blocks to induce *adaptation* contained the non-ambiguous continuum sounds that were congruent with the /aba/ or /ada/ video. Again, 10 blocks comprised exposure to “self” (5 Ab_self_Vb_self_ and 5 Ad_self_Vd_self_ blocks), and 10 blocks comprised exposure to “other” (5 Ab_other_Vb_other_ and 5 Ad_other_Vd_other_ blocks). The auditory test that followed exposure always contained speech sounds from the same speaker as seen and heard during the preceding AV exposure phase (i.e., “self” or “other”).

#### Immediate Capture

All 8 AV exposure stimuli (ambiguous/unambiguous audio × “self”/“other” × /aba/ or /ada/) were presented 10 times each in random order. After each AV stimulus, participants rated the perceived quality of the auditory part of the AV stimulus on a 7-point Likert-scale where “1” meant “clear /aba/” and “7” meant “clear /ada/.” Participants used the “1” through “7” keys on the standard keyboard to give their response. The next trial began 1000 ms after a response was collected.

## Results

In the auditory identification and the recalibration/adaptation tasks, we measured the proportion of “b”-responses. The corresponding analyses (repeated measures ANOVAs and *t*-tests, run in SPSS [version 20.0]) were conducted on the log odds transformed data, but we report proportions in the text and figures for reasons of clarity. The predictions described in the introduction do not necessarily assume a difference between “self” and “other,” and we therefore also conducted Bayesian repeated measures ANOVAs (in the JASP software, JASP Team, 2018) to bolster the interpretation of potential null-effects. Unlike conventional statistics, Bayesian statistics can be used to determine whether the data actually support the null-hypothesis (H0), or the alternative hypothesis (H1). To do so, the Bayes factor (BF_10_, or its inverse BF_01_) needs to be considered: when BF_10_ is larger than 3, the data are in favor of H1 (there is an effect or a difference), and when it is lower than the inverse (1/3, or 0.33), the data support H0. When BF_10_ is in between 0.33 and 3, the data are considered insensitive (Raftery, 1995; Wagenmakers, 2007).

### Auditory Identification

**Figure 2:**
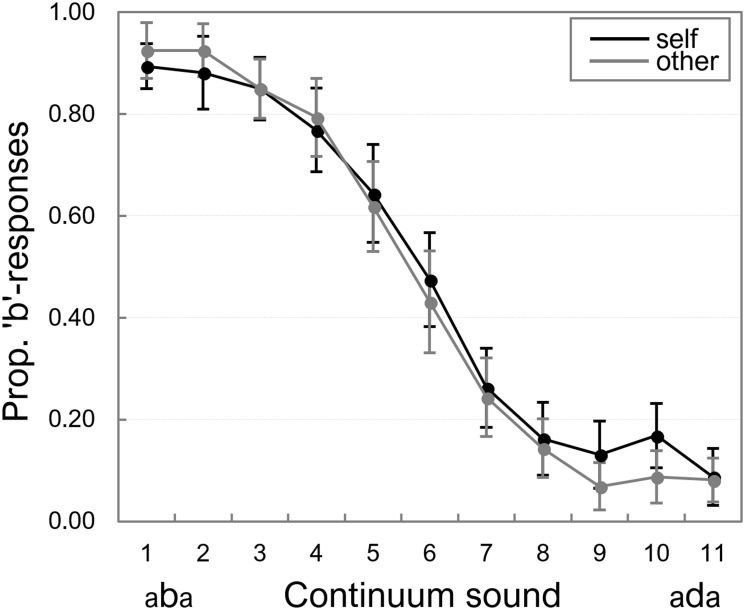
Proportion of “b”-responses on the auditory “self” and “other” continua. Error bars represent standard errors of the means.

Participants’ mean proportions of “b”-responses on each continuum token were calculated for the “self” and “other” separately. Figure 2 displays these proportions averaged across all participants.

A 2 (Speaker: “self” vs. “other”) × 11 (Continuum token) repeated measures ANOVA showed a main effect of Continuum token, *F*(10,150) = 81.65, *p* < 0.001, $\eta_{p}^{2}$ = 0.845, because the proportion of “b”-responses was higher for the most “b-like” tokens of the continua, and decreased as tokens became more “d-like.” There was no significant main effect of Speaker, *F*(1,15) < 1, and no significant interaction between the two factors, *F*(10,150) < 1^2^. We also fitted psychometric functions on the individual data (the proportion of “b”-responses), and the corresponding analyses (which confirmed that Speaker had no significant effect on identification of continuum tokens) are included in Appendix 1.

The Bayesian ANOVA supported the effect of Continuum token, as the ANOVA model with the main effect of Continuum token yielded a BF_10_ of 3.88e^+65^. The null-effect of Speaker was also supported: BF_10_ = 0.135 for the model that included only the main effect of Speaker, BF_10_ = 0.156 for the model that included both main effects, relative to the best model (with only the main effect of Continuum token), and BFs_10_ = 0.001 for the full model that included the interaction term, relative to the best model.

Taken together, these data thus indicate that the speech continua were perceived as intended, and identification of continuum tokens was not affected by whether participants heard themselves or someone else.

### Recalibration/Adaptation

**Figure 3:**
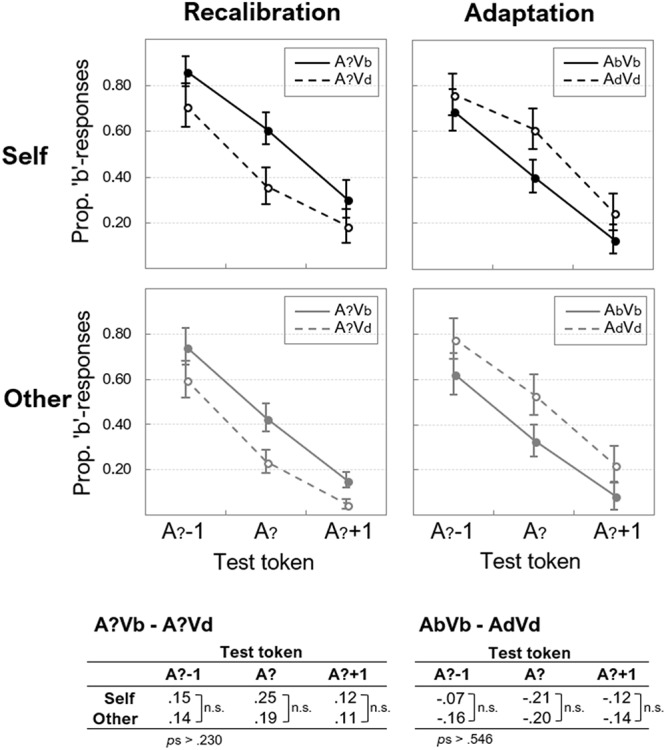
Auditory aftereffects after AV exposure. The proportion of “b”-responses (*y*-axes) are given for each test-token (*x*-axes) in the auditory test that followed exposure to ambiguous (left panels) and unambiguous (right panels) AV exposure stimuli, for “self” and “other.” Error bars represent standard errors of the means. The differences per token are provided below the panels, where recalibration is reflected by positive values, and adaptation by negative ones.

Participants’ mean proportions of “b”-responses on each auditory test token (A?-1, A?, and A?+1) were calculated, separately for “self” and “other” and for recalibration and adaptation. The averages across all participants are displayed in Figure 3.

An overall 2 (Speaker: “self” vs. “other”) × 2 (Adapter ambiguity: ambiguous vs. non-ambiguous) × 2 (Lip-read information: Vb vs. Vd) × 3 (Test token: A?-1, A?, A?+1) ANOVA showed no significant main effect of Speaker, *F*(1,15) = 2.73, *p* = 0.119, $\eta_{p}^{2}$ = 0.154, no main effect of Adapter ambiguity, *F*(1,15) < 1, and no main effect of Lip-read information, *F*(1,15) < 1. The main effect of Test token was significant, *F*(2,30) = 70.69, *p* < 0.001, $\eta_{p}^{2}$ = 0.825.

The interaction between Speaker and Adapter ambiguity approached significance, *F*(1,15) = 3.19, *p* = 0.067, $\eta_{p}^{2}$ = 0.207, because the proportion of “b” responses averaged across Vb and Vd adapters was somewhat higher for “self,” than for “other” (the difference was 0.14 for ambiguous adapters, and 0.05 for unambiguous ones). There was no interaction between Speaker and Lip-read information, *F*(1,15) < 1, and no interaction between Speaker and Test token, *F*(2,30) < 1.

Importantly, there was a significant interaction between Adapter ambiguity and Lip-read information, *F*(1,15) = 76.73, *p* < 0.001, $\eta_{p}^{2}$ = 0.836. As can be seen in Figure 3, this effect was observed because auditory aftereffects were modulated by Adapter ambiguity: for auditory ambiguous adapters, test responses were in accordance with the previously seen lip-read information (indicating *recalibration*), whereas for non-ambiguous adapters, test responses were more in accordance with the phonetic category opposite to what was seen and heard during exposure (indicating *adaptation*). Although the interaction between Adapter ambiguity and Test token was significant, *F*(30) = 4.10, *p* = 0.027, $\eta_{p}^{2}$ = 0.215, paired-samples *t*-tests revealed that actual differences between ambiguous or non-ambiguous exposure adapters per test token were not significant, *t*s(15) < 1.61, *p*s > 0.129. The interaction between Lip-read information and Test token was not significant, *F*(2,30) < 1.

The interaction between Speaker, Adapter ambiguity and Lip-read information was not significant, *F*(1,15) < 1, which was also the case for the interaction between Speaker, Adapter ambiguity and Test token, *F*(2,30) < 1, and the interaction between Speaker, Lip-read information and Test token, *F*(2,30) < 1.

The interaction between Adapter ambiguity, Lip-read information and Test token approached significance, *F*(2,30) = 2.87, *p* = 0.072, $\eta_{p}^{2}$ = 0.161, but adding the factor Speaker to this interaction yielded a non-significant effect, *F*(2,30) < 1.

Next, we quantified the recalibration and adaptation aftereffects as the difference between Vb and Vd (see Appendix 1 for individual aftereffects). Recalibration would thus yield *positive* aftereffects (i.e., A?Vb – A?Vd), whereas adaptation yields *negative* effects (AbVb – AdVd). As can be seen in Figure 3, this was indeed the case for all tokens, and these data were submitted to the Bayesian ANOVA (see Appendix 1 for model comparisons).

The data were most likely under the model that only included the main effect of Aftereffect (Recalibration vs. Adaptation), BF_10_ = 6.96e^+16^, and including Speaker as a main effect or as an interaction term yielded relative BFs_10_ < 0.206, supporting the null-effect of Speaker. The BF_10_ for the main effect of Test token (0.059) also supported H0, and adding Test token to any of the models that included Aftereffect, yielded BFs_10_ < 0.065 relative to the best model (i.e., the main effect of Aftereffect only).

These data thus showed that neither Speaker nor Test token had modulated the size of the recalibration and adaptation aftereffects. A one-sample *t*-test (against zero) on the recalibration effect pooled over Speaker and test token (0.16) showed that the effect was significantly larger than zero, *t*(15) = 4.67, *p* < 0.001, and likewise, the average adaptation effect (-0.15) was significantly smaller than zero, *t*(15) = 4.97, *p* < 0.001.

### Immediate Capture

**Figure 4:**
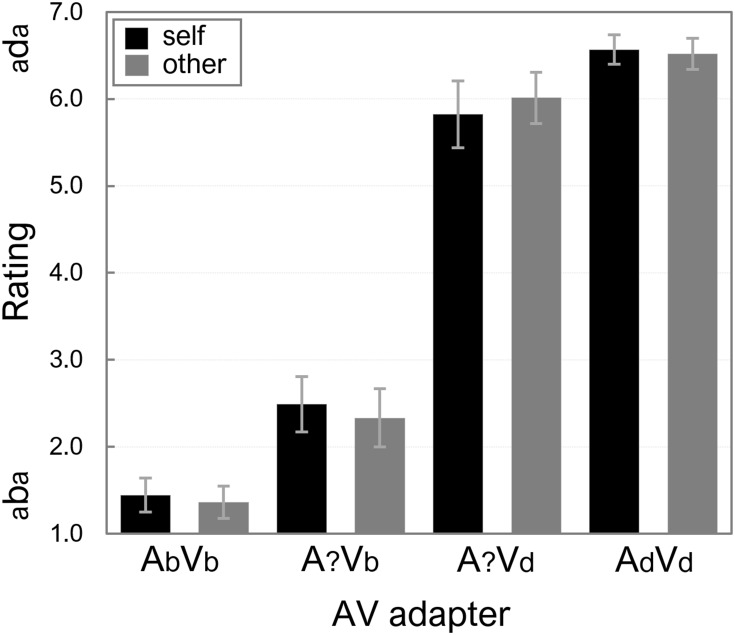
Mean ratings of the AV exposure stimuli for “self” and “other.” More “d-like” ratings are reflected by higher numbers on the *y*-axis. Error bars represent standard errors of the means.

Participants’ mean ratings on the AV exposure stimuli were calculated, and the averages across all participants are displayed in Figure 4.

A 2 (Speaker: “self” vs. “other”) × 2 (Adapter ambiguity: ambiguous vs. non-ambiguous) × 2 (Lip-read information: Vb vs. Vd) ANOVA on the ratings showed no significant main effects of Speaker and Adapter ambiguity, *F*s(1,15) < 1. There was a main effect of Lip-read information because the exposure adapters were rated more “aba-like” or “ada-like” depending on whether the lip-read information was /aba/ (mean rating was 1.91) or /ada/ (mean rating was 6.23).

The ANOVA also showed a significant interaction between Adapter ambiguity and Lip-read information, *F*(1,15) = 12.63, *p* = 0.003, $\eta_{p}^{2}$ = 0.457, because differences between mean ratings for adapters with unambiguous audio (1.40 vs. 6.54, for lip-read /aba/ vs. /ada/, respectively), as well as with ambiguous audio (2.41 vs. 5.92 for lip-read /aba/ vs. /ada/, respectively) were significant, *t*(15) > 7.93, *p*s < 0.001. The difference between A?Vb and A?Vd is most important here, because it reveals that the lip-read bias was similar for “self” and “other.”

None of the interaction effects that involved Speaker were significant, *F*s(1,15) < 1.29, *p*s > 0.274, and the null-effect of speaker was again supported by the Bayesian analyses (see Appendix 1 for model comparisons). The data were most likely under the model that included main effects of Lip-read information and Adapter ambiguity, and their interaction, BF_10_ = 3.43e^+45^. Adding Speaker as main effect, or including it in any interaction term yielded relative changes in BFs_10_ < 0.180. The importance of the factor Lip-read information was further highlighted by BFs_10_ < 0.229 in all models that excluded this factor.

## Discussion

We examined whether lip-read-induced effects on speech perception – manifested through immediate capture and recalibration – are modulated by whether participants were presented with stimulus materials that comprised their own voice and lip-read information (“self”), or someone else’s (“other”). Immediate capture and recalibration aftereffects were indeed observed, but there was no indication that these were modulated by “self” versus “other,” as supported by the parametric set of analyses and the Bayesian analyses that favored the null hypothesis in all models than involved Speaker (“self” vs. “other”). In the introduction, we highlighted previous work where a visual lip-read “self” advantage was observed with sentences (Tye-Murray et al., 2013, 2015) and an auditory voice “self” advantage was observed with McGurk pseudoword stimuli (Aruffo and Shore, 2012).

In both cases, the findings were discussed in a framework in which it was assumed that “self” generated stimuli are somehow stronger represented than stimuli generated by “others.” Aruffo and Shore (2012) argued that the “self” voice is perceived as more reliable than the “other” voice, and the “self” lip-read information therefore exerts a weaker influence on the percept than lip-read information for “other.” In our stimuli, we deliberately introduced uncertainty in the auditory speech signal to approximate real-life speech situations in which speech sounds are often ambiguous. As a result, lip-read information, and not the (“self”) voice became the most reliable source of information, and the percept was therefore adjusted toward the lip-read information, which resulted in the lip-read-induced immediate bias and the recalibration aftereffect.

Tye-Murray et al. (2013, 2015) argued that perception of the “self” lip-read signal produces a perceptual advantage because it shares a common code with some other domain, but it is not clear what this domain actually is. It could be the (planned actions of the) motor system – which is the most strict interpretation of the “common coding hypothesis” (e.g., Prinz, 1997) and aligns with the “motor theory of speech” (e.g., Liberman and Mattingly, 1985) –, it could consist of the lexico-semantic network that is involved during generation and perception of the sentences (which likely involves memory as well), or a combination of both. To complicate matters, lexical items in isolation (AV recordings of digits) do not produce a lip-read “self” advantage (Schwartz and Savariaux, 2001), and there is electrophysiological evidence for a lip-read “self” advantage for nonsense syllables (Treille et al., 2017). In that study, the authors observed that the lip-read-induced latency facilitation of the auditory N1 peak in the event-related potential – a tell-tale sign of audiovisual integration (e.g., van Wassenhove et al., 2005; Stekelenburg and Vroomen, 2007; Baart, 2016) – was stronger for “self” than for “other.” However, Treille et al. (2017) did not measure behavioral effects, so it is not clear if, and how, this early electrophysiological effect permeates down to the perceptual level.

Although we can only speculate about why different studies produced different findings, we do have a solid argument to explain why recalibration (and immediate capture) were similarly sized for “self” and “other.” From a functional perspective, it makes perfect sense that recalibration occurs for “other” as this is the default state of the world: we see and hear people talking to us, and we use the lip-read information to adjust for ambiguities in the sound if needed. The most straightforward explanation as to why this also occurs for “self” even though we hardly ever see and hear ourselves speaking, is that recalibration must be a general phenomenon.

Indeed, recalibration is quite robust and it occurs in many domains such as space (Radeau and Bertelson, 1974), time (Fujisaki et al., 2004; Vroomen et al., 2004) and emotional affect (Baart and Vroomen, 2018). It therefore seems that recalibration occurs whenever we are confronted with repeating, relatively small, cross-modal incongruities. Interestingly, however, when the audiovisual exposure videos clearly show that auditory ambiguities in the speech signal are caused by an external source (i.e., a pen in the mouth of the speaker), recalibration does not occur (Kraljic et al., 2008). This suggests that participants need to attribute the ambiguities in the auditory signal to the speaker rather than external incidental factors, before the perceptual system engages in recalibration. The current data extend this notion by showing that this is most likely independent of speaker identity: when ambiguities in the sound are perceived as idiosyncratic, it does not matter whether the speaker is “self” or “other,” as in both cases, similarly sized recalibration effects were observed.

For adaptation, we also found no difference between “self” and “other.” In general, when the auditory component of the stimulus was unambiguous, clear, and in correspondence with the lip-read information, the ambiguous test sounds were perceived more in accordance with the contrasting speech category: exposure to AbVb yielded more /ada/-responses during the test with ambiguous sounds, and exposure to AdVd yielded more /aba/-responses. For this adaptation effect, the reliability of the auditory signal relative to the lip-read signal is not critical [unlike in the work by Aruffo and Shore (2012)], because the effect is driven by the speech sound only, and not by the lip-read information that participants see Roberts and Summerfield (1981). The fact that we did not observe a difference between “self” and “other” therefore suggests that when the speech sound is completely clear (when participants repeatedly hear unambiguous /aba/ or /ada/), the “self” voice is not attributed more weight than the “other” voice, as both produced equal adaptation effects. These effects might be caused by the fact that repeated exposure to a clear speech sound (say, /aba/) causes fatigue of (hypothetical) linguistic feature detectors such that the ambiguous test sound is genuinely perceived as more “ada”-like (quite similar to color aftereffects driven by fatigue of retinal cells). However, it is also possible that adaptation reflects an acoustic contrast effect, which implies that the ambiguous sound is simply perceived as being different from the exposure sound, and therefore is categorized as belonging to the opposite phonetic category. We cannot disentangle these explanations, but it is clear that either the phonetic distance (related to the fatigue interpretation) or the acoustic distance (related to the contrast effect interpretation) between the unambiguous adaptation sound and the ambiguous test sound is a decisive factor in adaptation. This distance is the same for “self” and “other,” as were adaptation effects.

To conclude, we observed similar immediate lip-read capture, lip-read driven recalibration, and auditory driven adaptation for stimuli that comprised participants’ own talking face and voice, or someone else’s. The findings in the literature on lip-read and auditory “self” advantages in the speech domain are variable, and we did not observe any advantage for “self” over “other.” Perhaps, lip-read “self” advantages in other studies may have been related to lexico-semantic processes, which we minimized by using pseudowords. For recalibration, our findings are in-line with the notion that it reflects a domain-general learning mechanism that occurs whenever we are confronted with mild inter-sensory conflicts that, in the case of speech, cannot be attributed to external factors. For adaptation, our data suggest that the (acoustic or phonetic) distance between the clear exposure sound and the ambiguous test sound is critical, independent of the identity of the speaker.

## Author Contributions

MM, MP, and MB designed the experiments. MM and MB created the stimulus materials and analyzed the data. MM collected the data. All authors contributed to the writing process and agreed on the final text.

## Conflict of Interest Statement

The authors declare that the research was conducted in the absence of any commercial or financial relationships that could be construed as a potential conflict of interest.

## Footnotes

1 http://www.mattwinn.com/praat.html#formantContinuum

2 Reducing the number of levels for the factor Continuum token by only including five tokens from the middle ambiguous range (i.e., tokens 4 to 8) did not change the pattern of statistical significance.
