# Supplementary material for: No “Self” Advantage for Audiovisual Speech Aftereffects
Source: Front Psychol. 2019 Mar 22;10:658. doi: 10.3389/fpsyg.2019.00658 (PMC6440388; doi:10.3389/fpsyg.2019.00658)
Supplement: Supplementary file 1 [file Table_1.docx]

**Appendix 1**

We fitted individual logistic functions on the proportions of ‘b’-responses obtained in the auditory identification task for the ‘self’ and ‘other’. Next, we determined the individual ‘points of subjective equality’ (PSEs) for ‘self’ and ‘other’. The PSEs represent the perceptual middle points of the fitted curves that correspond to a proportion of ‘b’-responses of .5. Similarly, we computed the points on the curves that corresponded to proportions of ‘b’-responses of .75 and .25, and calculated the difference as an approximation to slope-steepness. There were no significant differences between ‘self’ and ‘other’, confirming that Speaker had not significantly affected auditory identification. The data and statistics are summarized in Table A1.

|  | Point on curve (Continuum token) | |
| --- | --- | --- |
|  | Self | Other |
| Proportion of ‘b’-responses of .75 | 4.25  ***t*(15) = 1.11, *p* = .285**  ***t*(15) = .389, *p* = .702**  ***t*(15) = .062, *p* = .952**  ***t*(15) = .201, *p* = .843** | 4.65 |
| Proportion of ‘b’-responses of .50 (PSE) | 5.69 | 5.59 |
| Proportion of ‘b’-responses of .25 | 7.14 | 6.52 |
| The .25 – .75 difference (approximation to slope steepness) | 2.89 | 1.87 |

***Table A1****: Averaged data and statistics for the fitted curves.*

The tables below provide the Bayesian model comparisons for the recalibration/adaptation task (table A2) and the immediate capture task (Table A3). The common observation is that the factor Speaker (‘self’ vs. ‘other’) never produced any evidence for H1. Instead, the main effects of Speaker support H0, and Speaker was never included in the models under which the data was most likely to occur. Adding Speaker to those models, always resulted in a relative change in BF_10_ smaller than .33, supporting the null-effect of Speaker.

***Table A2****: Model comparison for the 2 Speaker (self vs. other: coded as A) * 2 Aftereffect (recalibration vs. adaptation, coded as B) * 3 Test token (A?-1. A?, and A?+1, coded as C) Bayesian repeated measures ANOVA in the recalibration/adaptation task. The BF_10_ factor (< .33 supports a null-effect, > 3 supports H1) and error percentages are provided, and the model in bold represents the most likely model (i.e., with the highest BF_10_ factor) under which the data occurred. The column BF_10 change_ provides the change in BF_10_ for each model, relative to the best model.*

| Models | |  |  |  |  |  |  |  | BF_10_ | | error % | |  |  |  |  |  |  | BF_10 change_ |  |
| --- | --- | --- | --- | --- | --- | --- | --- | --- | --- | --- | --- | --- | --- | --- | --- | --- | --- | --- | --- | --- |
| A |  |  |  |  |  |  |  |  |  | .194 |  | 2.74 |  |  |  |  |  |  | 2.78e ^-18^ |  |
| **B** |  |  |  |  |  |  |  |  |  | **6.96e^+16^** |  | **.797** |  |  |  |  |  |  | - |  |
| A + B |  |  |  |  |  |  |  |  |  | 1.43e^+16^ |  | 1.26 |  |  |  |  |  |  | .205 |  |
| A + B + A  ×  B |  |  |  |  |  |  |  |  |  | 3.19e^+15^ |  | 4.58 |  |  |  |  |  |  | .046 |  |
| C |  |  |  |  |  |  |  |  |  | .059 |  | 1.41 |  |  |  |  |  |  | 8.48e ^-19^ |  |
| A + C |  |  |  |  |  |  |  |  |  | .012 |  | 6.90 |  |  |  |  |  |  | 1.72e ^-19^ |  |
| B + C |  |  |  |  |  |  |  |  |  | 4.51e^+15^ |  | 6.99 |  |  |  |  |  |  | .065 |  |
| A + B + C |  |  |  |  |  |  |  |  |  | 1.17e^+15^ |  | 22.2 |  |  |  |  |  |  | .017 |  |
| A + B + A  ×  B + C |  |  |  |  |  |  |  |  |  | 1.89e^+14^ |  | 2.21 |  |  |  |  |  |  | .003 |  |
| A + C + A  ×  C |  |  |  |  |  |  |  |  |  | .001 |  | 3.64 |  |  |  |  |  |  | 1.44e ^-20^ |  |
| A + B + C + A  ×  C |  |  |  |  |  |  |  |  |  | 1.09e^+14^ |  | 10.1 |  |  |  |  |  |  | .002 |  |
| A + B + A  ×  B + C + A  ×  C |  |  |  |  |  |  |  |  |  | 2.11e^+13^ |  | 3.03 |  |  |  |  |  |  | 3.03e ^-04^ |  |
| B + C + B  ×  C |  |  |  |  |  |  |  |  |  | 3.74e^+15^ |  | 2.82 |  |  |  |  |  |  | .054 |  |
| A + B + C + B  ×  C |  |  |  |  |  |  |  |  |  | 1.04e^+15^ |  | 26.3 |  |  |  |  |  |  | .015 |  |
| A + B + A  ×  B + C + B  ×  C |  |  |  |  |  |  |  |  |  | 1.66e^+14^ |  | 3.95 |  |  |  |  |  |  | .002 |  |
| A + B + C + A  ×  C + B  ×  C |  |  |  |  |  |  |  |  |  | 7.90e^+13^ |  | 2.48 |  |  |  |  |  |  | .001 |  |
| A + B + A  ×  B + C + A  ×  C + B  ×  C |  |  |  |  |  |  |  |  |  | 1.72e^+13^ |  | 2.91 |  |  |  |  |  |  | 2.47e ^-04^ |  |
| A + B + A  ×  B + C + A  ×  C + B  ×  C + A  ×  B  ×  C |  |  |  |  |  |  |  |  |  | 3.78e^+12^ |  | 2.15 |  |  |  |  |  |  | 5.43e ^-05^ |  |
| *All BF_10 change_ values < .33: adding factors did not significantly improve the best model* | | | | | | | | | | | | | | | | | | | |  |

***Table A3****: Model comparison for the 2 Speaker (self vs. other: coded as A) * 2 Adapter ambiguity (ambiguous vs. non-ambiguous, coded as B) * 2 Lip-read information (Vb vs. Vd, coded as C) Bayesian repeated measures ANOVA in the immediate capture task. The BF_10_ factor (< .33 supports a null-effect, > 3 supports H1) and error percentages are provided, and the model in bold represents the most likely model (i.e., with the highest BF_10_ factor) under which the data occurred. The column BF_10 change_ provides the change in BF_10_ for each model, relative to the best model.*

| Models | |  |  |  |  |  |  |  | BF_10_ | | error % | |  |  |  |  |  |  | BF_10 change_ |  |
| --- | --- | --- | --- | --- | --- | --- | --- | --- | --- | --- | --- | --- | --- | --- | --- | --- | --- | --- | --- | --- |
| A |  |  |  |  |  |  |  |  |  | .188 |  | .925 |  |  |  |  |  |  | 5.48e ^-47^ |  |
| B |  |  |  |  |  |  |  |  |  | .228 |  | 5.84 |  |  |  |  |  |  | 6.65e ^-47^ |  |
| A + B |  |  |  |  |  |  |  |  |  | .037 |  | 1.38 |  |  |  |  |  |  | 1.08e ^-47^ |  |
| A + B + A  ×  B |  |  |  |  |  |  |  |  |  | .011 |  | 7.16 |  |  |  |  |  |  | 3.21e ^-48^ |  |
| C |  |  |  |  |  |  |  |  |  | 6.24e ^+42^ |  | 5.20 |  |  |  |  |  |  | .002 |  |
| A + C |  |  |  |  |  |  |  |  |  | 1.07e ^+42^ |  | 1.51 |  |  |  |  |  |  | 3.12e ^-04^ |  |
| B + C |  |  |  |  |  |  |  |  |  | 1.64e^+42^ |  | 1.34 |  |  |  |  |  |  | 4.78e ^-04^ |  |
| A + B + C |  |  |  |  |  |  |  |  |  | 3.21e^+41^ |  | 3.09 |  |  |  |  |  |  | 9.36e ^-05^ |  |
| A + B + A  ×  B + C |  |  |  |  |  |  |  |  |  | 7.67e^+40^ |  | 2.80 |  |  |  |  |  |  | 2.24e ^-05^ |  |
| A + C + A  ×  C |  |  |  |  |  |  |  |  |  | 3.23e ^+41^ |  | 7.94 |  |  |  |  |  |  | 9.42e ^-05^ |  |
| A + B + C + A  ×  C |  |  |  |  |  |  |  |  |  | 9.60e^+40^ |  | 13.4 |  |  |  |  |  |  | 2.80e ^-05^ |  |
| A + B + A  ×  B + C + A  ×  C |  |  |  |  |  |  |  |  |  | 2.06e^+40^ |  | 2.45 |  |  |  |  |  |  | 6.01e ^-06^ |  |
| **B + C + B  ×  C** |  |  |  |  |  |  |  |  |  | **3.43e^+45^** |  | **2.71** |  |  |  |  |  |  | - |  |
| A + B + C + B  ×  C |  |  |  |  |  |  |  |  |  | 6.17e^+44^ |  | 2.81 |  |  |  |  |  |  | .180 |  |
| A + B + A  ×  B + C + B  ×  C |  |  |  |  |  |  |  |  |  | 1.89e^+44^ |  | 8.67 |  |  |  |  |  |  | .055 |  |
| A + B + C + A  ×  C + B  ×  C |  |  |  |  |  |  |  |  |  | 1.67e^+44^ |  | 2.54 |  |  |  |  |  |  | .049 |  |
| A + B + A  ×  B + C + A  ×  C + B  ×  C |  |  |  |  |  |  |  |  |  | 4.60e^+43^ |  | 3.62 |  |  |  |  |  |  | .013 |  |
| A + B + A  ×  B + C + A  ×  C + B  ×  C + A  ×  B  ×  C |  |  |  |  |  |  |  |  |  | 1.70e^+43^ |  | 4.18 |  |  |  |  |  |  | .005 |  |
| *All BF_10 change_ values < .33: adding factors did not significantly improve the best model* | | | | | | | | | | | | | | | | | | | |  |

Figure A1 displays the aftereffects per participant after exposure to ‘self’ and ‘other’ stimuli. The data on the left indicate *recalibration*, which is quantified as the difference between the proportion of ‘b’-responses after exposure to A?Vb and A?Vd, pooled over the three test-tokens (and mainly yields positive aftereffects). Likewise, the data on the right indicate *selective adaptation* (mainly negative aftereffects). Digits represent the number of participants for which a particular effect was observed.


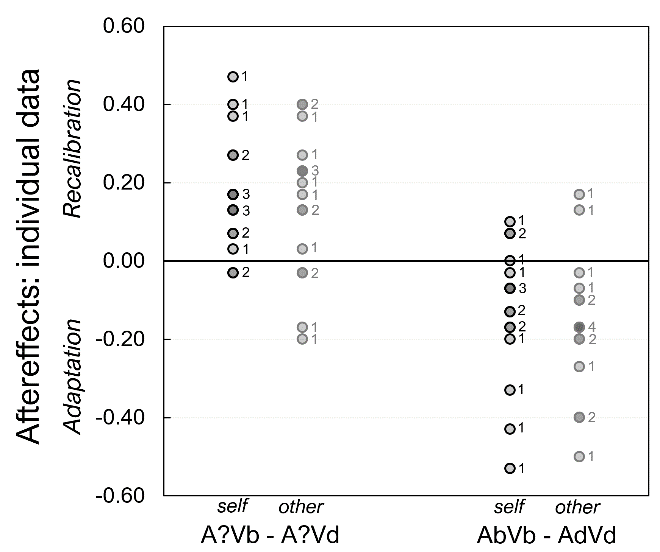
***Figure A1****: Individual aftereffects.*
